# Synergistic Effects of Cryotherapy and Radiotherapy in Glioblastoma Treatment: Evidence from a Murine Model

**DOI:** 10.3390/cancers17101692

**Published:** 2025-05-17

**Authors:** Hélène Cebula, Chrystelle Po, Carole Mura, Benoit Lhermitte, Roberto Luigi Cazzato, Marion Rame, Clara Le Fèvre, Julien Todeschi, Charles-Henry Mallereau, Afshin Gangi, Georges Noël, Michel de Mathelin, François Proust, Hélène Burckel

**Affiliations:** 1 Department of Neurosurgery, Hautepierre University Hospital, 1, Avenue Molière, 67200 Strasbourg, France; 2 Université de Strasbourg, CNRS, ICube UMR 7357, 67000 Strasbourg, France; po@unistra.fr (C.P.);; 3 Medicine Strasbourg University, 4 Rue Kirschleger, 67000 Strasbourg, France; 4 Radiobiology Laboratory, Institut de Cancérologie Strasbourg Europe (ICANS), UNICANCER, 17 Rue Albert Calmette, 67033 Strasbourg, France; 5 Pathology Department, University Hospital of Strasbourg, 67098 Strasbourg, France; 6 UMR CNRS 7021, Laboratory Bioimaging and Pathologies, OnKO3T Team, Faculty of Pharmacy, 67401 Illkirch, France; 7 Department of Interventional Radiology, University Hospital of Strasbourg, 1, Place de l’Hôpital, 67000 Strasbourg, France; 8 Department of Radiation Oncology, Institut de Cancérologie Strasbourg Europe (ICANS), UNICANCER, 17 Rue Albert Calmette, 67033 Strasbourg, France

**Keywords:** cryotherapy, radiotherapy, radiation, glioblastoma, apparent diffusion coefficient

## Abstract

Background/Objectives: Cryotherapy involves the insertion of cryoprobes into tumors to induce cell destruction through exposure to extremely low temperatures over several minutes. This localized treatment modality may enhance the efficacy of established therapies, such as radiotherapy, particularly for glioblastomas. Our study aimed to provide proof-of-concept for the efficacy of combining cryotherapy and radiotherapy in the treatment of subcutaneous murine brain tumors (GL-261) in immunocompetent C57BL/6 mice. Methods: Tumor growth, survival and response were evaluated using MRI and histological analysis. Results: Partial cryotherapy alone showed no therapeutic efficacy. However, combining cryotherapy with radiotherapy significantly potentiated treatment outcomes. A statistically significant survival benefit was observed in the combined therapy group compared to control, cryotherapy and radiotherapy groups. Notably, 40% of mice receiving the combined treatment exhibited complete responses, with no detectable tumor cells on MRI or histological analysis. Furthermore, MRI-based monitoring revealed that the Apparent Diffusion Coefficient (ADC) map could predict complete response 14 days post-treatment, unlike caliper-based measurements. Conclusions: These findings suggest that cryotherapy may enhance radiotherapy efficacy, resulting in complete tumor regression in 4 out of 10 cases. ADC distribution may serve as a predictive marker for therapeutic response. However, given the limitations of the model, further studies in orthotopic models are needed to validate these findings and assess their clinical relevance.

## Simple Summary

Glioblastoma is a highly aggressive brain tumor with a poor prognosis. Radiotherapy is a standard treatment, but its effectiveness is often limited. Cerebral cryotherapy, a technique involving controlled cooling of the tumor, has been shown to be safe in patients. This longitudinal study, using MRI (T2-weighted imaging and Apparent Diffusion Coefficient (ADC)) and histology, is the first to evaluate the combined effects of cryotherapy and radiotherapy in a glioblastoma mouse model. The results demonstrated a synergistic interaction between the two treatments, suggesting that their combination could improve therapeutic outcomes. Furthermore, ADC distribution was found to be a predictive marker of treatment response in this study. These findings could contribute to the development of more effective therapeutic strategies for glioblastoma, potentially advancing clinical practice and improving patient survival.

## 1. Introduction

Glioblastomas (GBM) represent the most common malignant primary tumors in adults with a median survival of 15–18 months [1]. The current standard of care at the time of GBM diagnosis involves a surgical resection followed by radiotherapy combined with concomitant temozolomide chemotherapy, and subsequent adjuvant chemotherapy. In some clinical settings, the concomitant use of tumor-treating fields (TTFields) is also recommended for patients who have completed the chemoradiotherapy regimen [2]. However, the recurrence of GBM is inevitable, with a median relapse-free survival limited to 8–9 months. No specific recommendation or guidelines exist for the management of recurrent GBM. The therapeutic options at this stage include surgical revision [3], radiotherapy [4] and novel chemotherapy agents [5,6]. The median progression-free survival after recurrence ranges from 1.5 and 4.3 months, with only 15% rate of survival at 6 months [5]. Other therapeutic approaches such as in situ chemotherapy [7], immunotherapy [8] and targeted therapies have been explored but failed to reach significant improvements in terms of overall survival. Additionally, focal therapies, including laser interstitial thermal therapy [9,10], TTFields [2], thermotherapy and photodynamic treatment have been developed but have yield similar outcomes [11].

Radiotherapy (RT) plays a central role in the management of GBM, both at initial diagnosis and at recurrence. Its primary mechanism of action involves the induction of DNA damage, particularly double strand breaks in tumor cells, leading to mitotic catastrophe, apoptosis, or senescence, depending on the tumor intrinsic repair capacity and the surrounding microenvironment. At the time of initial treatment, RT typically targets the tumor bed, residual tumor cells, and peritumoral edema following surgical resection [12]. The standard protocol involves fractionated irradiation, delivering a total dose of 60 Gy in 2 Gy fractions over six weeks, administered concurrently with temozolomide chemotherapy [1,13]. This combination reduces tumor burden and delays disease progression by enhancing tumor cell radiosensitivity and improving drug delivery, potentially through redistribution of the tumor vasculature. Despite its critical role, GBM exhibits substantial resistance to RT, driven by factors such as hypoxia, proficient DNA repair mechanisms, and pronounced intratumoral heterogeneity, which collectively limit the long-term effectiveness of conventional RT [14]. Upon recurrence, RT is typically applied to the progressive tumor regions within a limited volume [15], and efforts to enhance its efficacy through dose escalation, targeted delivery or combination with novel modalities are ongoing.

Currently, cryotherapy (CRYO) has emerged as an innovative treatment modality for various cancers such as lung, kidney, hepatic, bone and prostate [16,17]. As a focal therapy, cryotherapy holds promises for GBM treatment due to its unique properties. This technique involves the insertion of one or more cryoprobes into the tumor, exposing it to extremely low temperatures for several minutes. Cooling is achieved through the active circulation of argon gas within the probes, based on the Joules–Thomson principle [18]. Cryoablation is a precise and safe technique, based on the application of cycles of freezing and thawing phases to induce tumor destruction. Cryotherapy initiates four distinct injury mechanisms: direct and indirect cell injury, vascular damage and potential immumodulation [19]. While cryotherapy is intended to selectively target tumor vasculature to induce ischemia and necrosis, some collateral vascular damage to adjacent healthy tissues may occur, depending on the treatment parameters and the proximity of normal tissues to the tumor. This highlights the importance of precise tumor delineation during cryotherapy planning. This therapeutic approach alters several mechanisms that contribute to cancer survival, including cell proliferation, angiogenesis induction, tissue invasion, and immune response inhibition [20,21].

Given its underlying mechanisms, cryotherapy as a local treatment modality could be combined with existing therapies to enhance the efficacy of radiotherapy in GBM management. This proof-of-concept study aims to evaluate the efficacy of combined cryotherapy and radiotherapy compared to each treatment individually in a murine model of subcutaneously grafted brain tumors using immunocompetent mice.

## 2. Materials and Methods

### 2.1. Cell Culture

The mice glioma cell line GL-261 was purchased from the DSMZ Culture Collection (ACC 802). Cells were maintained at 37 °C in a humidified atmosphere with 5% CO_2_ and 95% air. GL-261 cells were cultured in DMEM (Dulbecco’s Modified Eagle Medium; Pan Biotech GmbH, Aidenbach, Germany) supplemented with 10% fetal calf serum (FCS, Pan Biotech GmbH) and 1% of penicillin-streptomycin solution (10,000 IU/mL penicillin and 10 mg/mL streptomycin; Pan Biotech GmbH). Subconfluent cell monolayers were trypsinized weekly using 0.05% trypsin containing 0.02% EDTA (Pan Biotech GmbH). Cell enumeration was conducted using a Countess^®^ Cell Counter (Invitrogen, Carlsbad, CA, USA).

### 2.2. Ethical Considerations and Animal Model

The study protocol was approved by ICANS institutional committee. All experimental procedures adhered to ethical guidelines and protocols and received approval from the French Ministry of Agriculture (approval numbers: #2019013010499402 and #2017082316306852). The experiments were conducted in accordance with the ARRIVE guidelines and relevant ethical standards.

GL-261 cells (10^6^) were suspended in 100 µL PBS/Matrigel^®^ (50/50; Corning^®^) and subcutaneously injected into the right flank of eight-week-old females C57BL/6J mice (Charles River, Écully, France). Twenty-seven days post-injection, mice with tumors measuring between 90 and 300 mm^3^ were randomly assigned into four groups: a non-treated control group (CTRL, n = 7); a cryotherapy group (CRYO, n = 11); a radiotherapy group (RT, n = 11) and a combined cryotherapy and radiotherapy group (CRYO-RT, n = 10). Tumor sizes were measured three times weekly using calipers, and the volume was calculated using the formula V = (Length × Width^2^)/2. Mice were monitored until the tumor volume reached 1500 m^3^ or for a maximum of 4 months post-inoculation. During cryotherapy, radiation treatment, and MRI monitoring, the animals were kept under anesthesia. Anesthesia was induced and maintained using a mixture of isoflurane and ambient air (2% for induction, 1.5% during the procedure). Mice were euthanized upon reaching a predefined endpoint or at a maximum of 120 days post-treatment using an overdose of anesthesia (5% isoflurane in 100% oxygen), followed by cervical dislocation, in accordance with ethical guidelines.

Animals were randomly assigned to treatment groups, and investigators conducting MRI analysis, histological evaluation, and outcome assessments were blinded to treatment allocation.

### 2.3. Cryotherapy

Partial cryotherapy was conducted using a 4 mm cryoablation probe (Freezor 207F, 7F diameter, Medtronic^®^, Meerbusch, Germany) and a CryoConsole system (Medtronic^®^). The tumor area was shaved and following an incision, the cryoprobe was inserted into the center of the tumor mass. The tumor was then exposed to two freezing cycles, each lasting 20 s at −50 °C, separated by a 20 s thawing cycle. A predefined iceball volume (6 × 4 mm) was determined prior to the procedure to ensure treatment of only a portion of the tumor, allowing for the evaluation of the effect on the residual tumor tissue. To prevent hypothermia, the mice were positioned on a heated pad during the cryotherapy treatment, and the tumor surface was warmed using a hot water glove immediately following cryotherapy. All procedures were performed under strictly sterile conditions. The control and radiotherapy groups of mice also underwent an incision and subcutaneous insertion of a cryoprobe to mimic the tissue damage caused by the insertion of the cryotherapy probe into the tumor.

### 2.4. Irradiation

Irradiation was delivered 24 h after cryotherapy with a single dose of 10 Gy of X-rays (Primus, 6 MeV, Siemens, Erlangen, Germany) at a dose rate of 200 UM/min. The 24 h interval between cryotherapy and radiotherapy was intentionally selected to assess skin tolerance and ensure the absence of cryotherapy-induced necrosis before initiating irradiation. During irradiation, the mouse was positioned on a heated mat and placed beneath a lead shield (Xraystore), allowing selective exposure of the tumor-bearing right leg to the radiation beam.

### 2.5. Magnetic Resonance Imaging

Each mouse underwent Magnetic Resonance Imaging (MRI) one day prior to treatment (except for CTRL group), followed by MRIs one day post-treatment, and then every 15 days thereafter until euthanasia. The latter occurred either when the tumor volume reached 1500 m^3^ or after a maximum of 4 months post-inoculation. A last MRI exam was performed just before the euthanasia. The control group followed the same procedure except for the pretreatment MRI. Mice were positioned in an MRI compatible heated cradle (Minerve) with warm air. Their respiratory rate was monitored via a breathing pillow placed under the thorax. The breathing rate was maintained at 90–110 breaths/min by adjusting the isoflurane concentration. Vital functions were continuously monitored during the entire anesthesia period using a Physioguard system (Minerve).

MRI experiments were conducted on a 7T Bruker Biospec horizontal MR System (Bruker Biospin). Radiofrequency transmission and reception were achieved using a Bruker quadrature volume resonator (inner diameter: 86 mm) and a single-loop surface coil (diameter: 20 mm), respectively. Initial fast anatomical images of the tumor were acquired with transversal relaxation time-weighted axial images (T2WI) using a Rapid Acquisition with Relaxation Enhancement (RARE) sequence (TR/TE: 2500/33 ms, RARE factor: 9, 2 averages, 22 slices contiguous, resolution: 78 × 78 × 700 μm^3^, TA: 3 min). Diffusion-weighted images (DWI) were acquired in a single direction assuming isotropic water diffusion in the tumor, using a diffusion-weighted spin-echo sequence (TR/TE: 2500/24 ms; NA: 1, TA: 24 min, Δ/δ: 11/5 ms, 3 b-values: 0, 500 and 1000 s/mm^2^, 22 slices contiguous, resolution: 78 × 78 × 700 μm^3^). Finally, high-resolution anatomical images were obtained using a RARE sequence (TR/TE: 3550/42.6 ms, RARE factor: 9, 30 slices contiguous, resolution: 78 × 78 × 300 μm^3^, TA: 18 min).

Tumor volumes were manually segmented by two independent experimenters using 3DSlicer version 4.11, based on anatomical images corrected for bias (N4bias correction, ANTs) and smoothed using a Gaussian filter [22]. The signal intensity (SI) of the T2WI in the tumor area and its associated standard deviation (SD) were measured from bias-corrected anatomical images and masked using FMRIB Software Library (version 6.0) [23]. Tumor heterogeneity was determined as the ratio of SD to SI. An Apparent Diffusion Coefficient (ADC) map was generated from DWI data using MRtrix3 software (version 3.0.2) [24]. A tumor mask was applied to the ADC maps to extract the individual histograms and the mean ADC and SD values for each tumor. From these histograms, individual µADC values were calculated using a simple or double Gaussian fit (Curve Fitter, Matlab, version R2023B, Natick, MA, USA).

### 2.6. Histology and Immunofluorescence Analysis

Mice were euthanized when tumors reached a maximum volume of 1500 mm^3^ or after four months of post-treatment survival. The tumors, or the corresponding tissue area if no tumor was detected, were excised and fixed in 4% formalin for 24 h before paraffin embedding. Tissue blocks were sectioned at a thickness of 4 microns. Slides were stained with hematoxylin and eosin (H&E) using an automated staining system (Leika, Nußloch, Germany). Additional sections were prepared for the immunohistochemical analysis of GFAP and Ki67 expression. For GFAP and Ki67 staining, slides were deparaffinized, rehydrated, and subjected to antigen retrieval using a citrate buffer (pH 6.0) at 95 °C for 10 min. Endogenous peroxidase activity was quenched using 0.5% hydrogen peroxide in methanol for 30 min at room temperature. Slides were then incubated with primary antibodies against GFAP (dilution: 1:200, Cell Signaling, Danvers, MA, USA) and Ki67 (dilution: 1:200, Cell Signaling) at 4 °C overnight. After washing, slides were incubated with biotinylated secondary antibodies, then extravidin peroxydase and visualized using a diaminobenzidine chromogen system. Counterstaining was performed with hematoxylin, and slides were dehydrated and coverslipped. Staining was evaluated under a light microscope to assess GFAP expression in glial cells and Ki67 as a marker of cellular proliferation.

### 2.7. Statistical Analysis

All figures and statistical analysis were performed using GraphPad Prism (version 10.1.0). Statistical significance was defined as *p*-value ≤ 0.05. Kaplan–Meier curves were generated for tumor growth experiments, and log-rank tests were assessed to compare the survival outcomes between two treatment groups.

## 3. Results

### 3.1. Tumor Monitoring Using Caliper Measurements and MRI: There Is an Impact of Combining Cryotherapy and Radiotherapy

**Figure 1:**
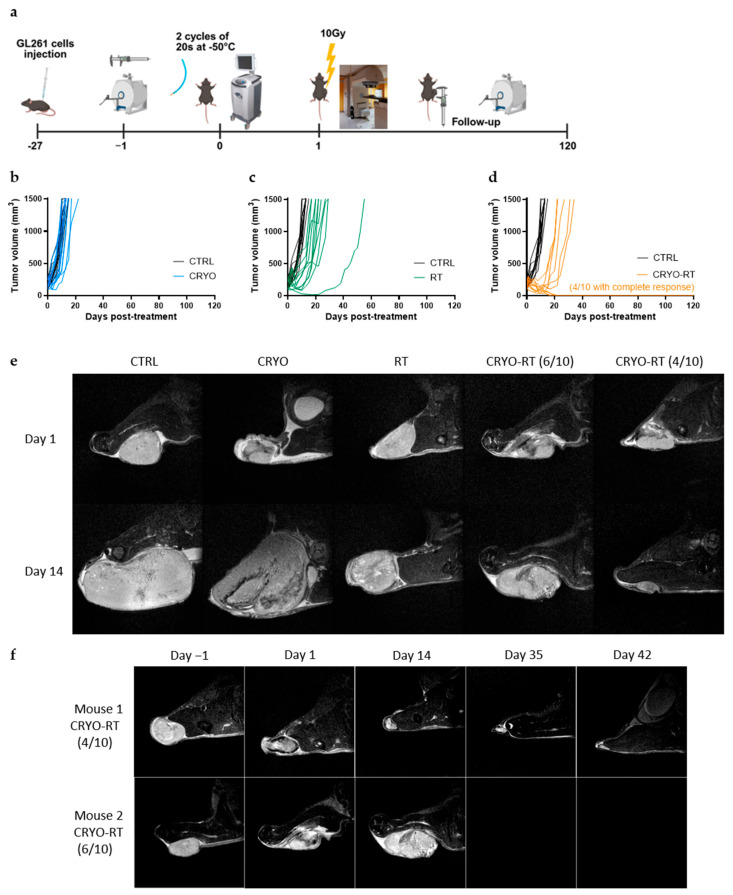
Tumor volume progression over time (−1 to 120 days post-treatment) in response to different treatments modalities. (**a**) Overview of experimental timeline and tumor growth (created in BioRender): (**b**) cryotherapy (CRYO); (**c**) a single 10 Gy dose of radiation (RT); and (**d**) the combination of cryotherapy with 10 Gy radiation (CRYO-RT), compared to the untreated sham control group (CRTL). GL-261 GBM cells were injected subcutaneously into C57BL/6J mice on day −27, and tumor growth was monitored over 120 days post-treatment. (**e**) MRI images illustrating tumor growth progression at 1 and 14 days post-treatments for CTRL, CRYO, RT, CRYO-RT groups, including those reaching the volume threshold (6/10) and those in complete response (4/10) following CRYO-RT treatment. (**f**) Longitudinal MRI-based assessment of tumor volume progression in CRYO-RT–treated mice from day 1 to day 42 post-treatment. Mouse 1, representative of 4 out of 10 mice in the CRYO-RT group, exhibited complete tumor regression with non-palpable tumors observed from day 20 onward. In contrast, Mouse 2, representative of the remaining six mice, showed progressive tumor growth, reaching the maximum allowed volume of 1500 mm^3^.

Tumor growth was monitored three times a week using calipers until the tumor volume reached 1500 mm^3^ or a maximum of four months post-inoculation (Figure 1a–e). Given our objective to assess the combined therapeutic effect of RT and CRYO, partial cryotherapy was intentionally performed in order to preserve residual tumor tissue for subsequent irradiation. Irreversible tumor cell damage is typically achieved at temperatures ranging from −40 °C and −50 °C, a range widely employed in oncologic cryotherapy procedures to ensure effective ablation, while minimizing collateral damage to surrounding healthy tissue. Accordingly, cryotherapy was applied at −50 °C in this study. The progression of tumor volume over time within each group demonstrated similar growth trends between the control (CTRL) and the cryotherapy (CRYO) groups, illustrating that partial cryotherapy had no significant impact on tumor progression (Figure 1b and Figure S1a–d). In contrast, a delay in tumor growth was observed in the radiotherapy group (RT, Figure 1c) and was even more pronounced in the combined radiotherapy and cryotherapy group (CRYO-RT, Figure 1d). The combined CRYO-RT group demonstrated a greater delay in tumor growth than other treatments, displaying two distinct response profiles (Figure 1f). The evolution of the tumor volume estimated from T2WI was similar (Figure 1e,f).

Notably, in four out of ten mice in the combined CRYO-RT group, non-palpable tumors were detected as early as 20 days post-treatment (Figure 1d–f). In the case of non-palpable tumors, MRI enabled the visualization of deeper anatomical structures at day 14, confirming the absence of detectable tumors (Figure 1e,f). We further explored the correlation between baseline tumor volume and treatment response. The graphical representation (Figure S1e) reveals no clear association, as both small and large tumors were observed to either regress completely or progress to the predefined tumor volume threshold. These findings indicate that baseline tumor size does not influence treatment efficacy in this model.

### 3.2. Mice Survival: A Significantly Increased Survival Was Observed in CRYO-RT Group Compared to Other Treatment Groups

**Figure 2:**
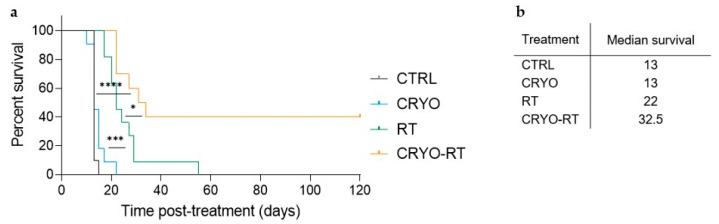
Survival outcomes following treatments with cryotherapy (CRYO, blue), a single 10 Gy dose of radiation (RT, green), and the combination of cryotherapy with 10 Gy radiation (CRYO-RT, orange), compared to the sham control group (CRTL, black). (**a**) Kaplan–Meier survival curves for C57BL/6 mice bearing subcutaneous GL-261 xenografts, treated with CRYO, RT, CRYO-RT or left untreated (CTRL). Mice were monitored for survival up to 120 days post-treatment. Statistical significance was determined using the log-rank test, with results indicated as follows: * *p* < 0.05 for comparisons between RT and CRYO-RT groups, *** *p* < 0.001 for comparisons between CRYO and RT groups and **** *p* < 0.0001 for comparisons between CRYO and CRYO-RT groups. (**b**) Median survival times (in days post-treatment) for each treatment group.

In terms of the treatment’s efficacy evaluation, Kaplan–Meier survival curves were plotted (Figure 2a). Kaplan–Meier survival analysis revealed no statistically significant difference between the CTRL and CRYO groups, both exhibiting a median survival of 13 days (Figure 2a,b). In comparison, a significant improvement in survival was observed in the RT group compared to the CTRL and CRYO groups (*p* < 0.001). The CRYO-RT group demonstrated significantly longer survival compared to the RT group, with median survival times of 32.5 days and 22 days, respectively, demonstrating a statistically significant difference (*p* < 0.05). Additionally, four out of ten mice in the CRYO-RT group achieved a complete response, with no detectable tumors confirmed on MRI, and survived beyond 120 days.

### 3.3. MRI Analysis: Prediction of Treatment Effectiveness

#### 3.3.1. Tumor Heterogeneity

**Figure 3:**
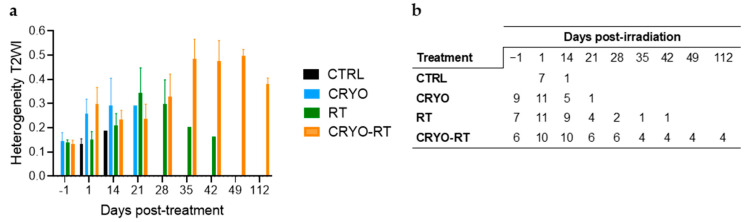
(**a**) Evolution of signal intensity heterogeneity (T2-weighted imaging (T2WI) signal) in the tumor area according to different treatment groups, including sham control (CRTL), cryotherapy (CRYO), a single 10 Gy dose of radiation (RT), and the combination of cryotherapy with 10 Gy radiation (CRYO-RT). (**b**) Number of surviving animals per group based on post-irradiation time in each group.

In addition to assessing tumor volume, the anatomical images showed heterogeneity in signal intensity in the tumor area (Figure 3a,b). The cryotherapy groups (CRYO and CRYO-RT) exhibited increased heterogeneity in T2-weighted imaging (T2WI) signal intensity compared to the CTRL group on day 1. By day 14, signal heterogeneity in both cryotherapy groups stabilized, while it exhibited a slight increase in the RT and CTRL groups. Notably, heterogeneity in the CRYO-RT group decreased over this period, approaching levels observed in the RT group by days 14 and 21. By day 28, signal heterogeneity in the RT and the CRYO-RT groups had stabilized at comparable levels. However, from day 35, heterogeneity in the CRYO-RT group increased, while that in the RT group began to decline.

#### 3.3.2. Evolution of Mean ADC Values

**Figure 4:**
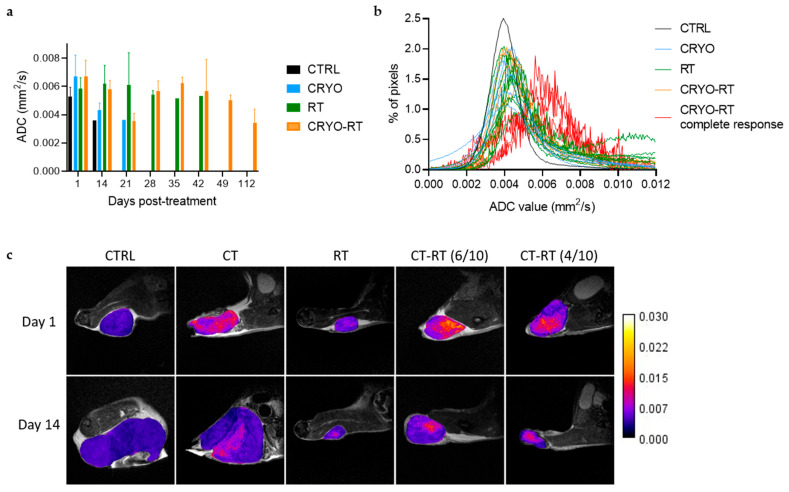
Evolution of mean apparent diffusion coefficient (ADC) values (in mm^2^/s) within the tumor area across control (CRTL), cryotherapy (CRYO), single 10 Gy dose of radiation (RT), and combined cryotherapy with 10 Gy radiation (CRYO-RT) treatment groups. (**a**) Evolution of mean ADC maps in all groups from day 1 to 112 post-treatment. (**b**) Individual ADC histograms of the tumor area at day 14 post-treatment, highlighting the four mice out of ten in the CRYO-RT group with non-palpable tumors at day 20 (red, complete response). (**c**) MRI anatomical images illustrating differences in ADC among all treatment groups (CTRL, CRYO, RT, CRYO-RT (6/10) and CRYO-RT (4/10)) between days 1 and 14.

The evolution of mean apparent diffusion coefficient (ADC) values in tumor areas was assessed across treatment groups (Figure 4a). One day post-treatment, ADC values were increased in the cryotherapy-treated groups (CRYO and CRYO-RT), although this elevation was not statistically significant. From days 1 to 21, ADC values remained comparable between these two groups despite contrasting tumor growth. Tumors in the CRYO group continued to grow, whereas in the CRYO-RT group, tumors either slowed in growth or decreased in volume. Both ADC maps and anatomical images showed heterogeneity in the tumor tissue at 1 and 14 days post-treatment (Figure 4a–c). In contrast, no significant change in ADC was observed in the RT group during this period. Between days 14 and 21, a decline in ADC was noted in the CRYO group. By day 28, ADC values in the RT and the CRYO-RT groups converged. Subsequently, starting on day 35, ADC values in the RT group decreased; however, data were limited due to the presence of only one remaining mouse in this group. In contrast, ADC values in the CRYO-RT group increased and stabilized between days 35 and 112 post-treatment.

The ADC value histograms in the tumor for individual mice on day 14 allowed the identification of four mice in CRYO-RT group. For these four mice, tumors remained non-palpable from day 20, in contrast to the other mice, that were sacrificed once their tumor reached 1500 mm^3^ (Figure 4b). Indeed, a shift in the ADC curves was observed for these four animals compared to the others (Figure 4b, red curves). The peak of the histogram, representing the mean ADC (µADC) extracted from curve fitting, was significantly higher in these 4 mice (µADC 0.006 ± 0.0004 mm^2^/s) compared to the remaining animals, regardless of the treatment (µADC 0.004 ± 0.0003 mm^2^/s) (Figure 4b).

### 3.4. Histological Analysis: Confirmation of Complete Response

**Figure 5:**
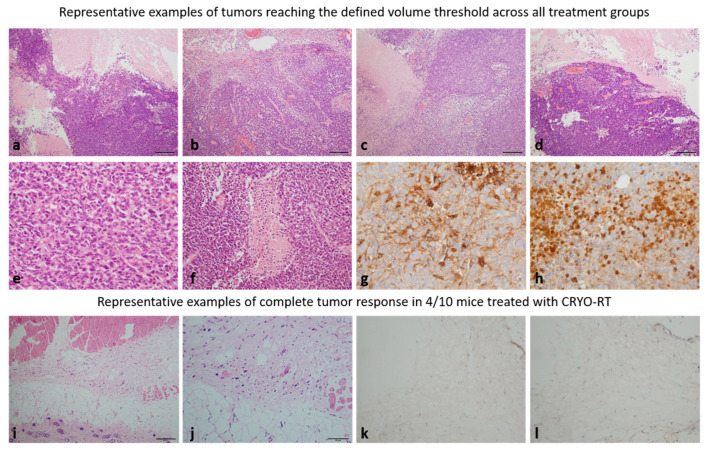
Hematoxylin and eosin staining, along with GFAP and Ki67 markers analyzed in tumor tissue from different groups: (**a**) untreated control (CTRL), (**b**) cryotherapy (CRYO), (**c**) a single 10 Gy dose of radiation (RT), (**d**) the combination of cryotherapy and 10 Gy radiation (CRYO-RT). For the CTRL, CRYO, RT and some CRYO-RT groups, staining was performed when the tumor volume reached 1500 m^3^ (bar scale 50 µm). Representative example of a tumor, irrespective of treatment group, that reached the defined volume threshold, displaying numerous mitotic figures (×40) (**e**) and characteristic geographic areas of necrosis (×20) (**f**). (**g**) Representative example of a tumor, irrespective of treatment group, that reached the defined volume threshold, showing focal expression of GFAP (×20). (**h**) In the same tumor, the Ki67 proliferation index was estimated to be approximately 40% (×20). (**i**,**j**) Staining in mice with non-palpable tumors from the CRYO-RT group was performed four months post-inoculation (bar scale 50 µm). (**k**) Subcutaneous tissue in non-palpable tumors is focally thickened by fibrosis, with no GFAP-positive cells observed (×20). (**l**) The Ki67 proliferation index in non-palpable tumors is zero (×20).

Histological examination of the tumors with hematoxylin-eosin staining revealed no significant differences among the different groups: CTRL (Figure 5a), CRYO (Figure 5b), RT (Figure 5c), and CRYO-RT (Figure 5d). All tumors presented densely cellular architecture. The tumor cells presented with oval or irregularly shaped hyperchromatic nuclei and relatively scant basophilic cytoplasm. Numerous mitotic figures were observed (Figure 5e), along with regularly present geographic necrotic areas (Figure 5f).

Immunohistochemical analysis indicated that, irrespective of the treatment group, all tumors that reached a limited volume threshold (>1500 mm^3^) consistently expressed glial fibrillary acid protein (GFAP), a marker of glial origin (Figure 5g). Additionally, the proliferation marker Ki67 was estimated to be at 40% in these tumors (Figure 5h).

In the case of the four mice exhibiting a complete response (Figure 5i–l), no tumor was detected. Instead, a fibrotic area was observed at the interface of the hypodermis and striated muscle tissue, characterized by the presence of a few cells displaying hyperchromatic nuclei and poorly defined cytoplasm (Figure 5i,j). Notably, no mitotic activity was observed in this area. Additionally, both Ki67 and GFAP were negative in these mice (Figure 5k,l).

## 4. Discussion

In this study, we aimed to assess the therapeutic potential of cryotherapy both as a monotherapy and in combination with radiotherapy. Previous research has demonstrated that cryotherapy can induce a protective effect when applied to the entire tumor, followed by the reinjection of GL261 tumor cells into the treated area [25]. Consequently, we deliberately avoided complete tumor cryotherapy in our approach, which revealed a significant synergistic effect between cryotherapy and radiotherapy. This combination thereby presents a novel therapeutic strategy for glioblastoma that warrants further exploration.

Our findings revealed a statistically significant improvement in survival in the group receiving combined cryotherapy and radiotherapy compared to the control, cryotherapy and radiotherapy groups. Partial cryotherapy of the tumors did not provide any therapeutic benefit. Notably, within the combined cryotherapy and radiotherapy (CRYO-RT) group, four animals achieved complete responses, as confirmed by MRI and histological analysis, with no detectable residual tumor cells. Longitudinal MRI monitoring, particularly ADC mapping, predicted complete responses as early as 14 days post-treatment, whereas tumor volume measurements using T2WI or caliper-based techniques failed in detecting early responses.

Although partial cryotherapy alone was ineffective, its combination with radiotherapy resulted in significant therapeutic effects. The mechanisms behind this observed potentiation of radiotherapy by cryotherapy likely involve several factors. Indeed, cryotherapy induces necrosis in the frozen tumor area and causes endothelial damage and apoptosis in the surrounding tissues [21]. Cryotherapy, as a local therapeutic modality, potentiates the efficacy of radiotherapy, as evidenced by the pronounced delay in survival growth and the observed and confirmed complete responses. The therapeutic efficacy of cryotherapy is attributed to the sequential phases of rapid freezing followed by slow thawing. This cyclic freezing process causes severe hypothermia in the targeted tissue, resulting in both direct and indirect cellular damage. Specifically, the formation of the iceball in the cells leads to membrane rupture, and during thawing, vasoconstriction of larger micro-arterioles (>500 µm) and the obstruction of smaller vessels exerts a direct effect on endothelial cells and contribute to ischemia [21]. Steinbach et al. have demonstrated that cryotherapy induces apoptosis at a cellular periphery [26].

We observed variability in treatment responses within the CRYO-RT group, with 4 out of 10 mice achieving a complete response, while 6 out of 10 reached the 1500 mm^3^ tumor volume threshold. This variability is further emphasized by the lack of correlation between baseline tumor volume and treatment response. These findings underscore the need for further investigation to identify the underlying factors contributing to differential responses, potentially through the use of more refined models. Radioresistance, may arise from factors inherent to the tumor microenvironment, including tumor heterogeneity, hypoxia, metabolic alterations (such as enhanced glycolysis), DNA repair mechanisms, microRNA expression, and alterations of apoptotic pathways, is a well-known challenge in GBM [27]. The administration of radiosensitizers could enhance the efficacy of radiotherapy without increasing radiation doses [28]. Cryotherapy itself may act as a radiosensitizer by disrupting neovascularization, thereby reducing hypoxia or inducing tumor cell apoptosis [19]. For example, Huang et al. demonstrated DNA fragmentation associated with cryotherapy and a synergistic effect with recombinant human tumor necrosis factor-alpha (rhTNF-α) in promoting cellular apoptosis [29]. Other potential mechanisms may involve altered cellular membrane integrity and reduced DNA repair capacity, particularly in the lipid bilayer [28]. However, the exact mechanisms by which cryotherapy potentiates radiotherapy remain to be fully elucidated, and further studies are required to directly investigate vascular, apoptotic, and immune changes associated with this combination therapy.

Regarding imaging, we investigated the heterogeneity of T2WI signal intensity within the tumor. T2WI signal heterogeneity has been proposed as a non-invasive imaging biomarker of malignancy in various tumor types, including GBM [30,31,32].This imaging biomarker is influenced by various biological processes, including necrosis, inflammation, fibrosis, cyst formation, hemorrhage, and variations in cellular density. Typically, necrosis and edema are associated with increased T2 signal intensity, while hemorrhage, increased cellular density, and gliosis tend to reduce T2 signal intensity. Although T2WI signal heterogeneity reflects the tumor’s cellular and microenvironment diversity, we did not observe a correlation between T2WI signal heterogeneity and treatment response in our study.

We also analyzed the ADC maps of tumors, a non-invasive biomarker indicative of cellular density. ADC values are inversely correlated with cell density: lower ADC values typically correspond to regions with high tumor cellularity, while higher values suggest decreased cellular density, often due to necrosis or inflammation [33]. Monitoring changes in ADC values over time can therefore provide valuable insights into tumor biology and treatment response. Following treatment, an increase in ADC was observed, suggesting reduced cellular density, likely due to tumor necrosis or inflammatory responses. One day post-treatment, the cryotherapy groups (CRYO and CRYO-RT) presented higher mean ADC values compared to other groups, which was supported by increased T2 heterogeneity. These findings may indicate an inflammatory response, consistent with cryotherapy’s known immune-modulating effects. Previous studies have shown that cryotherapy induces dendritic cell maturation and activation, thereby enhancing their antigen-presenting function [19,20]. Fourteen days post-treatment, a distinct ADC distribution was observed in only four mice from the CRYO-RT group, which later achieved complete responses starting at 20 days post-treatment as assessed by caliper measurements. Notably, these four mice displayed higher µADC values compared to others, possibly indicating tumor cell death due to the synergic effects of cryotherapy and radiotherapy. Our results suggest that ADC may serve as a predictive marker of therapeutic response in this model [34,35]. This finding aligns with the proposal that ADC could serve as a predictive biomarker for treatment response in GBM in clinical settings [36].

Our study presents several limitations. Although the complete responses observed in 4 out of 10 mice are promising, the unforeseen variability in treatment outcomes within the combination treatment group, along with the limited sample size, reduces the robustness and generalizability of our conclusions. A subcutaneous GBM model was employed due to the large size of the available cryotherapy probes, which precluded their application in an orthotopic intracerebral setting in murine models. Consequently, this model does not accurately replicate the GBM microenvironment, including the presence and influence of the blood–brain barrier (BBB), immune microenvironment, and tumor-host interactions, all of which may influence treatment efficacy. While this model allows for precise monitoring and intervention, it lacks the complexity of an orthotopic model. The main objective was to evaluate the feasibility of combining cryotherapy and radiotherapy in a preclinical GBM model. However, we now recognize the need for future studies using orthotopic models to more accurately evaluate the translational relevance of these therapeutic strategies and their potential for clinical application.

Importantly, a key mechanism that warrants further investigation is the potential disruption of the BBB induced by cryotherapy. One of our separate clinical studies has shown that cryoablation for recurrent GBM can transiently disrupt the BBB, allowing better delivery of systemic therapies [37]. The integrity of the BBB plays a critical role in the molecular resistance mechanisms of GBM to therapeutic agents. Several strategies have been developed to transiently enhance BBB permeability, with ultrasound-mediated microbubble injection demonstrating promising results in preclinical models [38]. Therefore, future research should use alternative animal models bearing orthotopic GBM tumors that can accommodate the cryoprobe to further elucidate the mechanisms underlying the combined effects of cryotherapy and radiotherapy.

Cryotherapy induces ischemia and necrosis in both tumors and surrounding tissues, with the extent of tissue damage depending on the treatment parameters and the tumor’s proximity to healthy tissues. In our patient cohort treated with cryotherapy for recurrent GBM, we demonstrated both the feasibility and safety of this approach [39]. MRI-guided cryotherapy enabled real-time monitoring of iceball formation, allowing for complete coverage of the tumor recurrence, while minimizing damage to surrounding healthy tissue. The iceball formation is clearly visualized in real time on MRI, with well-defined and precise margins, facilitating accurate targeting of the tumor area.

Given GBM’s highly infiltrative nature, which often involves critical brain regions, cryotherapy alone may pose risks when applied near sensitive structures. The iceball formation delivers ablative temperatures to both tumor and adjacent tissues, but its inability to conform precisely to irregular tumor shapes limits its applicability near critical structures. Radiotherapy, particularly stereotactic radiosurgery (SRS) or fractionated radiotherapy, offers highly conformal dose distributions that can selectively target tumor margins and infiltrative extensions, while sparing adjacent organs at risk. Therefore, combining cryotherapy with stereotactic or fractionated RT could provide a complementary approach: cryotherapy would ablate the tumor core, while RT could target peripheral infiltration zones or tumor regions near sensitive structures that are less amenable to cryoablation. Furthermore, cryotherapy may act as a radiosensitizing agent, enhancing the efficacy of subsequent RT. Future studies should explore the integration of cryotherapy with precision radiotherapy techniques, such as SRS or proton therapy, to optimize tumor control while minimizing collateral damage to healthy tissues.

## 5. Conclusions

This proof-of-concept study suggests that cryotherapy may enhance the therapeutic efficacy of radiotherapy in GBM, potentially through mechanisms such as disruption of the blood–brain barrier. While these findings are based on a subcutaneous tumor model and should be interpreted with caution, it is noteworthy that cryotherapy has already been tested in patients with recurrent GBM, demonstrating both safety and feasibility. These prior clinical results, along with our preclinical data, highlight the potential of cryotherapy as a complementary modality to the current standard-of-care, including the Stupp protocol. However, further studies in orthotopic models and with larger sample sizes are needed to validate these findings and to better understand the underlying mechanisms before translating this strategy into clinical practice.

## Data Availability

All data referenced in this manuscript are available from the corresponding author upon request.

## Supplementary Materials

The following supporting information can be downloaded at: https://www.mdpi.com/article/10.3390/cancers17101692/s1, Figure S1: Individual tumor volume progression over time across treatment groups: (a) control (CTRL), (b) cryotherapy (CRYO), (c) a single 10 Gy dose of radiotherapy (RT) and (d) combined cryotherapy with 10 Gy radiotherapy (CRYO-RT). In panel (d), mice are further categorized based on treatment response, with orange curves representing those reaching the maximum allowable tumor volume threshold (6/10) and red curves indicating complete responders (4/10). (e) Baseline tumor volume versus treatment outcome. Each point represents an individual mouse at treatment initiation, plotted according to its baseline tumor volume. Mice were stratified into two groups based on treatment outcome: complete responders (n = 4, red) which exhibited full tumor regression, and progressers (n = 6, orange) which reached the predefined tumor volume limit. This graphical representation shows that both small and large baseline tumor volumes were present in both groups, indicating no correlation between initial tumor size and treatment outcome.

## Abbreviations

The following abbreviations are used in this manuscript:

ADC	Apparent Diffusion Coefficient
CRYO	Cryotherapy
CRYO-RT	Cryotherapy and radiotherapy
CTRL	Control
GBM	Glioblastoma
GFAP	Glial fibrillary acid protein
MRI	Magnetic Resonance Imaging
RT	Radiotherapy
T2WI	Transversal relaxation time-weighted axial images
