# Supplementary material for: Synergistic Effects of Cryotherapy and Radiotherapy in Glioblastoma Treatment: Evidence from a Murine Model
Source: Cancers (Basel). 2025 May 17;17(10):1692. doi: 10.3390/cancers17101692 (PMC12110222; doi:10.3390/cancers17101692)
Supplement: Supplementary file 1 [file cancers-17-01692-s001.zip › cancers-3610232-supplementary.pdf]

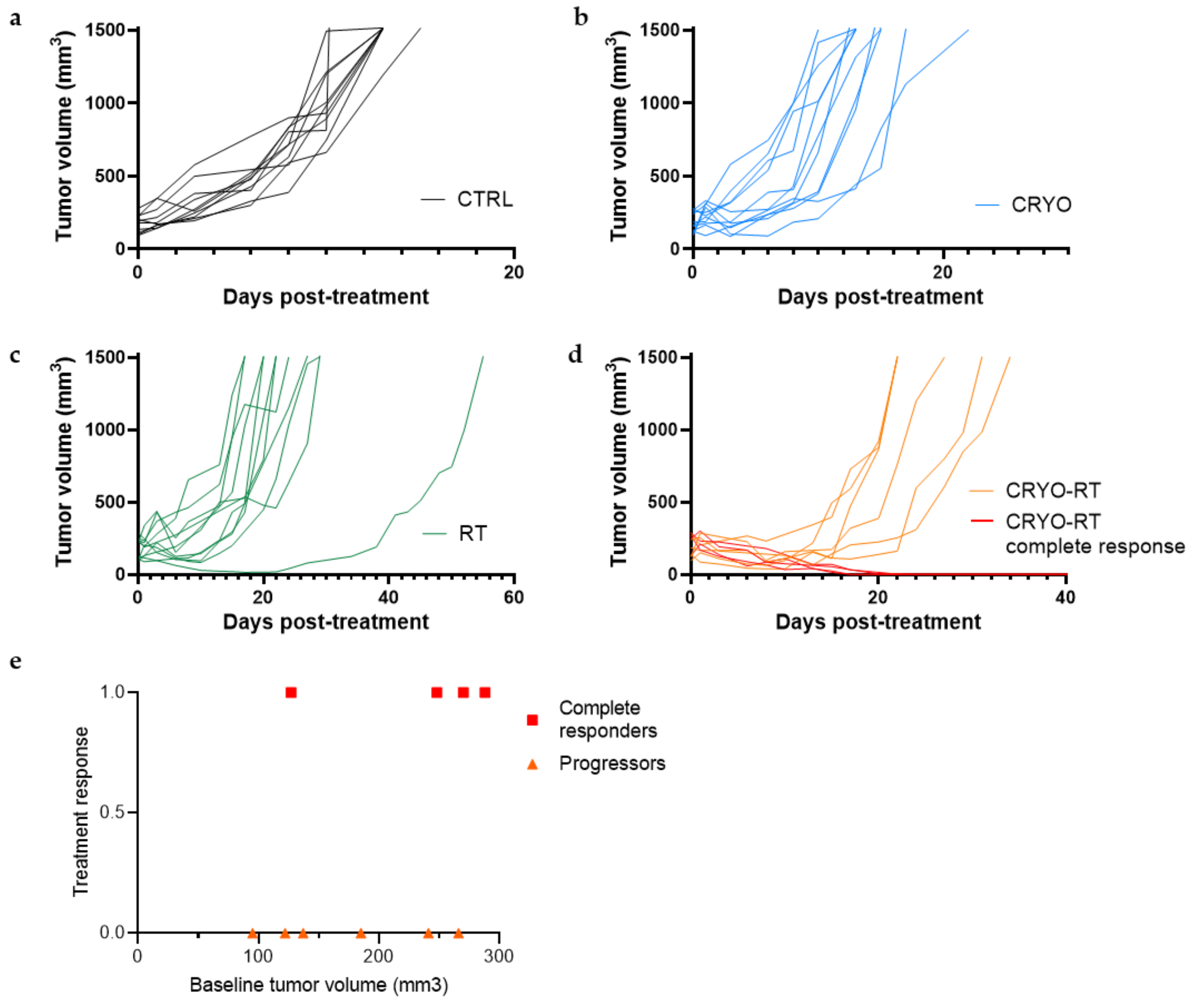

Supplementary Figure S1. Individual tumor volume progression over time across treatment groups: (**a**) control (CTRL), (**b**) cryotherapy (CRYO), (**c**) a single 10 Gy dose of radiotherapy (RT) and (**d**) combined cryotherapy with 10 Gy radiotherapy (CRYO-RT). In panel (**d**), mice are further categorized based on treatment response, with orange curves representing those reaching the maximum allowable tumor volume threshold (6/10) and red curves indicating complete responders (4/10). (**e**) Baseline tumor volume versus treatment outcome. Each point represents an individual mouse at treatment initiation, plotted according to its baseline tumor volume. Mice were stratified into two groups based on treatment outcome: complete responders (n = 4, red) which exhibited full tumor regression, and progressers (n = 6, orange) which reached the predefined tumor volume limit. This graphical representaion shows that both small and large baseline tumor volumes were present in both groups, indicating no correlation between initial tumor size and treatment outcome.
